# The vitamin D receptor gene as a determinant of survival in pancreatic cancer patients: Genomic analysis and experimental validation

**DOI:** 10.1371/journal.pone.0202272

**Published:** 2018-08-14

**Authors:** Federico Innocenti, Kouros Owzar, Chen Jiang, Amy S. Etheridge, Raluca Gordân, Alexander B. Sibley, Flora Mulkey, Donna Niedzwiecki, Dylan Glubb, Nicole Neel, Mark S. Talamonti, David J. Bentrem, Eric Seiser, Jen Jen Yeh, Katherine Van Loon, Howard McLeod, Mark J. Ratain, Hedy L. Kindler, Alan P. Venook, Yusuke Nakamura, Michiaki Kubo, Gloria M. Petersen, William R. Bamlet, Robert R. McWilliams

**Affiliations:** 1 UNC Eshelman School of Pharmacy, University of North Carolina at Chapel Hill, Lineberger Comprehensive Cancer Center, Chapel Hill, North Carolina, United States of America; 2 Department of Biostatistics and Bioinformatics, Duke University Medical Center, Durham, North Carolina, United States of America; 3 Duke Cancer Institute, Duke University Medical Center, Durham, North Carolina, United States of America; 4 North Shore University Health System, Evanston, IL, United States of America; 5 Northwestern University, Chicago, IL, United States of America; 6 University of California at San Francisco, San Francisco, CA, United States of America; 7 Moffitt Cancer Center, Tampa, FL, United States of America; 8 University of Chicago, Chicago, IL, United States of America; 9 Center for Genomic Medicine, RIKEN, Yokohama, Japan; 10 Mayo Clinic, Rochester, MN, United States of America; German Cancer Research Center, GERMANY

## Abstract

**Purpose:**

Advanced pancreatic cancer is a highly refractory disease almost always associated with survival of little more than a year. New interventions based on novel targets are needed. We aim to identify new genetic determinants of overall survival (OS) in patients after treatment with gemcitabine using genome-wide screens of germline DNA. We aim also to support these findings with in vitro functional analysis.

**Patients and methods:**

Genome-wide screens of germline DNA in two independent cohorts of pancreatic cancer patients (from the Cancer and Leukemia Group B (CALGB) 80303 and the Mayo Clinic) were used to select new genes associated with OS. The vitamin D receptor gene (VDR) was selected, and the interactions of genetic variation in VDR with circulating vitamin D levels and gemcitabine treatment were evaluated. Functional effects of common VDR variants were also evaluated in experimental assays in human cell lines.

**Results:**

The rs2853564 variant in *VDR* was associated with OS in patients from both the Mayo Clinic (HR 0.81, 95% CI 0.70–0.94, p = 0.0059) and CALGB 80303 (HR 0.74, 0.63–0.87, p = 0.0002). rs2853564 interacted with high pre-treatment levels of 25-hydroxyvitamin D (25(OH)D, a measure of endogenous vitamin D) (p = 0.0079 for interaction) and with gemcitabine treatment (p = 0.024 for interaction) to confer increased OS. rs2853564 increased transcriptional activity in luciferase assays and reduced the binding of the IRF4 transcription factor.

**Conclusion:**

Our findings propose *VDR* as a novel determinant of survival in advanced pancreatic cancer patients. Common functional variation in this gene might interact with endogenous vitamin D and gemcitabine treatment to determine improved patient survival. These results support evidence for a modulatory role of the vitamin D pathway for the survival of advanced pancreatic cancer patients.

## Introduction

Advanced pancreatic cancer is rarely curable at the time of diagnosis and is typically associated with survival of little more than a year. Estimated new cases of advanced pancreatic cancer in the USA for 2017 is 53,670 and an estimated number of deaths at 43,090 [1]. Recent approved therapies conferring modest survival advantages include folfirinox, nab-paclitaxel, and others. Currently, no biomarkers are available to predict response in patients with this disease [2].

Multidisciplinary efforts have focused on improving tools for early diagnosis and cataloging the architecture of somatic mutations. In conjunction with these efforts, the interrogation of the germline genome of patients [3] aims to characterize the constitutive basis of biological systems of fundamental importance for tumor biology, such as angiogenesis, inflammation, immunity, and others [4]. Scanning the entire genome for germline DNA variants results in the discovery of novel candidate genes worthy of further investigation [5].

In the present study, our aim was to discover and validate novel genes and pathways of importance in the biology of pancreatic cancer and in the pharmacology of its treatment. We employed data from two genomic screens of germline DNA to identify variants associated with survival. Starting with single-nucleotide polymorphisms (SNPs) associated with survival from the Cancer and Leukemia Group B (CALGB) 80303 trial, we incorporated another cohort of pancreatic cancer patients from the Mayo Clinic where a genome-wide screen was conducted previously [6]. We then identified SNPs with the same effect in both datasets (CALGB 80303 and Mayo Clinic) to pinpoint genes of clinical relevance for the outcome of patients, and on which to conduct molecular and cellular studies.

## Patient and methods

### CALGB 80303: patient population, genotyping, and measurement of 25-hydroxyvitamin D (25(OH)D)

CALGB 80303 was a double-blind, placebo-controlled, randomized phase III trial of gemcitabine, in combination with bevacizumab, in advanced pancreatic adenocarcinoma patients. Genome-wide genotype data of 330,690 directly interrogated SNPs were collected from 294 genetically-estimated European patients using the Illumina 550K platform (S1 Fig). The characteristics of the patients enrolled in the genome-wide screen are described in Table 1. Overall survival (OS) is defined as the time from registration to death from any cause. The median follow-up time was 36 months. Patient eligibility, characteristics, stratifications, response evaluation, treatments, and quality control of the genotyping have been described previously [7]. This research has been approved by the Institutional Review Board of each participating institution and all patients provided written informed consent. Serum levels of 25(OH)D were previously measured at baseline (at the time of study registration) by an FDA-approved immunoassay [8]. CALGB is now part of the Alliance for Clinical Trials in Oncology.

**Table 1:** Clinical characteristics of patients from the CALGB 80303 and Mayo Clinic studies.

		CALGB 80303	Mayo Clinic
Number of patients		294	408
**Sex**	Male	159 (54.1%)	233 (57.1%)
	Female	135 (45.9%)	175 (42.9%)
**Age**	Median (95% CI)	64.0 (63.0, 65.5)	67.5 (67.0, 69.0)
**Extent of disease**	Metastatic	256 (87.1%)	198 (48.5%)
	Locally advanced	38 (12.9%)	209 (51.2%)
	Missing		1 (0.3%)
**Prior radiotherapy**	No	262 (89.1%)	279 (68.4%)
	Yes	32 (10.9%)	119 (29.1%)
	Missing		10 (2.5%)
**Performance status**	ECOG 0 or 1	265 (90.1%)	-
	2	29 (9.9%)	-
	Karnofsky 90	-	100 (24.5%)
	Karnofsky 80	-	146 (35.8%)
	Karnofsky 70	-	111 (27.2%)
	Karnofsky 50 and 60	-	42 (10.3%)
	Missing		9 (2.2%)
**Treatment**	Gemcitabine/Placebo	140 (47.6%)	-
	Gemcitabine/Bevacizumab	154 (52.4%)	-
	Gemcitabine	-	203 (56.4%)
	Other chemotherapy	-	46 (11.3%)
	Information not available	-	58 (14.2%)
	No chemotherapy	-	74 (18.1%)
**Overall survival time**	Median (months) (95% CI)	5.95 (5.36, 6.97)	8.05 (7.69, 8.90)

Patients from these two studies are genetically-estimated Europeans. Ninety-six percent of the Mayo Clinic patients had histological confirmation of pancreatic cancer, with the remainder meeting pre-specified criteria for a clinical diagnosis of pancreatic adenocarcinoma (must have pancreatic mass with/without metastases, and clinical syndrome of weight loss, painless jaundice, and/or pain, +/- serum CA19-9 >100 U/mL, reviewed by a medical oncologist with subspecialty expertise in pancreatic cancer).

### Mayo Clinic: Patient population and genotyping

Four hundred eight genetically-estimated European patients with advanced pancreatic adenocarcinoma were recruited at the Mayo Clinic (Table 1). Recruitment, consent and specimen collection were described previously [6]. Patient records were reviewed by a subspecialist in gastrointestinal malignancy and confirmed to represent a diagnosis of pancreatic adenocarcinoma. All participating patients completed a risk factor questionnaire at enrollment that included self-reported Karnofsky performance score, along with lifestyle and family history information. Staging was performed at study entry using the American Joint Committee on Cancer 6^th^ edition criteria. OS was defined as the time elapsed from date of the first visit to the Mayo Clinic for pancreatic cancer diagnosis (or treatment) to date of death. In order to minimize lead-time bias, patients were excluded if their first visit to the Mayo Clinic was more than 3 months after their initial diagnosis of cancer. The median follow-up time was 87 months. Death dates were obtained from online sources (Accurint®), death certificates, medical records, or family communication.

As with the CALGB 80303 cohort, genotyping was performed using the Illumina 550K platform. Quality control of the 551,766 SNPs used in this study was previously described [6] (S1 Fig). The Institutional Review Board of the Mayo Clinic approved this study.

### Statistical analysis

The primary objective of this study was to select novel candidate genes associated with OS using genomic screens from two cohorts of advanced pancreatic cancer patients. The candidate genes were further investigated for their clinical relevance and the mechanistic basis linking them to OS.

In CALGB 80303, the association with OS was tested using the Cox proportional hazards model under an additive genetic model. The SNPs were ranked by score (log-rank) p value for their association with OS, and the top 300 SNPs (an arbitrary cut-off) were selected to be tested for association with OS in the Mayo Clinic cohort. The same statistical methods were used in this group of patients as CALGB 80303, and the Wald test p values are reported. Note that the score test was used for CALGB 80303 as it typically performs better in univariate analyses, though the two tests are asymptotically equivalent.

After testing the 300 SNPs for association with OS in the Mayo Clinic patients, the following filtering criteria were used to prioritize SNPs and genes for further investigation: 1) a SNP must be located within a gene (10 Kb flanking the 3’ and 5’ ends), 2) have p<0.05 for association with OS, and 3) the minor frequency allele should have the same direction of effect on OS in both studies. SNPs meeting these criteria were considered concordant between the two cohorts. Note that here the p value cutoff of <0.05 was used merely for feature selection for further analysis, and hence has not been adjusted for multiple comparisons.

The associations between SNPs and OS were first analyzed without adjusting covariates. Then, as a sensitivity analysis of the robustness of the genetic associations, SNPs underwent additional testing for their associations with OS by including prognostic cofactors in the Cox proportional-hazards for each data set. Analyses of the Mayo Clinic data were adjusted for disease stage, Karnofsky performance score, age, and body-mass index, while analyses of the CALGB 80303 data were adjusted for study stratification factors and ancestry (3 principal components of variance based on the genetic European study population).

The interaction between rs2853564 and either serum levels of 25(OH)D or gemcitabine treatment was tested using a multiplicative Cox proportional hazards model, i.e., the model included a term for the product of the two variables. The interaction with gemcitabine treatment was only possible in the Mayo Clinic cohort, as a group of patients was not treated with any chemotherapy. The p value of the interaction term indicates whether the effect on OS of either 25(OH)D or gemcitabine treatment differs among the genotypes. The interaction analyses were not adjusted for any covariates. Serum levels of 25(OH)D were tested as a continuous measure, however, for illustrative purposes, Kaplan-Meier plots and median survival times are provided using a previously established cut-off of 21.7 ng/ml [8] to define high and low levels of 25(OH)D.

### Function of the VDR SNPs in luciferase reporter assays

rs2853564 was tested for its functional effects on the transcriptional activity of *VDR*. rs7979131 was also selected because of its near-perfect linkage disequilibrium (LD) with rs2853564 and putative functionality based upon bioinformatics (S1 Table). A 1.8 kb region of *VDR* encompassing both variants was PCR amplified and cloned upstream of a minimal promoter in pGL4.26[luc2/minP/Hygro], which contains the *Firefly* luciferase reporter gene. Human pancreatic carcinoma cells (PANC-1) and telomerase-immortalized human microvascular endothelial (TIME) cells were used. More experimental details are provided in the Supplementary Materials and Methods section.

### Electrophoretic mobility shift assays (EMSA) of VDR SNPs

rs2853564 and rs7979131 are both intronic SNPs of *VDR* which might alter the binding of transcription factors. Based upon the bioinformatics analysis, IRF4 and SPI1 are two putative transcription factors with binding sites proximal to rs2853564 (7 and 13 bp, respectively), while the binding site of CTCF spans rs7979131 (S2 Table). Biotinylated DNA probes containing either the reference sequence or variant alleles were synthesized for each SNP and were incubated with over-expression cell lysates for either IRF4 or SPI1 (both for rs2853564), and CTCF (for rs7979131). Shift analyses were carried out using the LightShift Chemiluminescent EMSA kit according to the manufacturer’s instructions.

### Association between VDR rs2853564 and VDR mRNA expression in pancreatic cancer tissues and cell lines

To determine whether rs2853564 is associated with mRNA levels of *VDR* in tumors and cancer cell lines, 66 resected primary pancreatic adenocarcinomas from a separate cohort of patients [9] (S3 Table) and 44 pancreatic cancer cell lines were examined. These methods are described in the Supplementary materials and methods.

## Results

### Associations between SNPs and OS in two cohorts of pancreatic cancer patients

The top 300 most significant SNPs associated with OS in CALGB 80303 were tested for association in patients from the Mayo Clinic. This design was intended to identify SNPs that had a concordant effect between the two datasets (i.e., unadjusted p value <0.05 and same direction of effect) and to select SNPs and genes for further analysis. Of the 300 SNPs, 10 had similar effects on OS between the two studies and a p value <0.05 in the unadjusted analysis (Table 2). Four SNPs were located in known genes (*VDR*, *CMYA5*, *C7orf58*, *CAMK4*). Of the four genes, the analysis of the literature linking them to pancreatic cancer indicated a role for *VDR* and the vitamin D pathway. [10–14] Hence we have analyzed the association between the genetic variation in *VDR* and OS, and its interaction with patient vitamin D levels and gemcitabine treatment. We furthermore prioritized this genetic variation in *VDR* for in vitro experimental testing to validate our findings.

**Table 2:** SNPs with concordant effects and p<0.05 in the genome-wide screens of both the CALGB 80303 and Mayo Clinic patients.

							CALGB 80303			Mayo Clinic		
SNP (rsid)	Ch	Gene	Feature	5’ flanking gene	3’ flanking gene	Base change	MAF	Unadj. HR (95% Cl) Adj. HR (95% Cl)	Unadj. p value Adj. p value	MAF	Unadj. HR (95%CI) Adj. HR (95%CI)	Unadj. p value Adj. p value
10500873	11	NA	NA	*DBX1*	*HTATIP2*	A>G	0.031	0.43 (0.26–0.69) 0.45 (0.28–0.73)	0.0004 0.0012	0.053	0.67 (0.51–0.88) 0.61 (0.45–0.81)	0.0046 0.0009
**2853564**	**12**	***VDR***	**intron**	***LOC728166***	***TMEM106C***	**A>G**	**0.406**	**0.74 (0.63–0.87)** **0.71 (0.60–0.83)**	**0.0002** 3.7*10^**−5**^	**0.390**	**0.81 (0.70–0.94)** **0.81 (0.70–0.94)**	**0.0059** **0.0059**
1566122	8	NA	NA	*LPL*	*SLC18A1*	G>A	0.307	0.71 (0.60–0.85) 0.72 (0.60–0.86)	0.0002 0.0003	0.339	0.82 (0.70–0.96) 0.82 (0.70–0.96)	0.0113 0.0134
10107943	8	NA	NA	*LPL*	*SLC18A1*	G>A	0.310	0.70 (0.58–0.83) 0.70 (0.59–0.84)	6.7*10^−5^ 0.0001	0.339	0.82 (0.70–0.96) 0.82 (0.70–0.96)	0.0128 0.0151
2169430	16	NA	NA	*LOC642659*	*hCG_2045437*	G>A	0.303	0.73 (0.61–0.87) 0.77 (0.64–0.93)	0.0006 0.0069	0.319	0.82 (0.70–0.96) 0.85 (0.72–1.00)	0.0127 0.0479
259095	5	*CMYA5*	intron	*PAPD4*	*LOC100132835*	A>G	0.053	0.50 (0.35–0.74) 0.50 (0.34–0.74)	0.0003 0.0005	0.052	0.69 (0.50–0.95) 0.63 (0.45–0.87)	0.0226 0.0057
989978	10	NA	NA	*NKX2-3*	*SLC25A28*	G>A	0.255	0.71 (0.59–0.86) 0.73 (0.60–0.89)	0.0005 0.0022	0.282	0.85 (0.74–0.99) 0.89 (0.77–1.04)	0.0358 0.1369
2126337	16	NA	NA	*LOC642659*	*hCG_2045437*	A>G	0.364	0.72 (0.61–0.86) 0.77 (0.65–0.92)	0.0001 0.0030	0.396	0.86 (0.74–0.99) 0.85(0.73–0.98)	0.0327 0.0259
1534016	7	*C7orf58*	intron	*ING3*	*WNT16*	A>G	0.376	0.74 (0.62–0.88) 0.74 (0.62–0.89)	0.0006 0.0009	0.368	0.86 (0.75–1.00) 0.84 (0.73–0.98)	0.0477 0.0227
306104	5	*CAMK4*	intron	*LOC402287*	*STARD4*	A>G	0.347	0.68 (0.56–0.81) 0.67 (0.56–0.81)	2.3*10^−5^ 2.6*10^−5^	0.356	0.86 (0.73–1.00) 0.89 (0.76–1.05)	0.0496 0.1675

The results for rs2853564 in *VDR* are shown in bold. Cox score (log-rank) p values are reported for CALGB 80303, and Wald p values are reported for the Mayo Clinic data. Adjusted (Adj) results are for associations adjusted for several variables (see Methods). Unadjusted (Unadj) results are for associations not adjusted for any variables. Note that the adjusting variables vary between the two patient cohorts due to different study designs and limitations in the data collected. Ch, chromosomal location; MAF, relative minor allele frequency.

### Association between VDR rs2853564 and OS in two cohorts of pancreatic cancer patients

**Fig 1:**
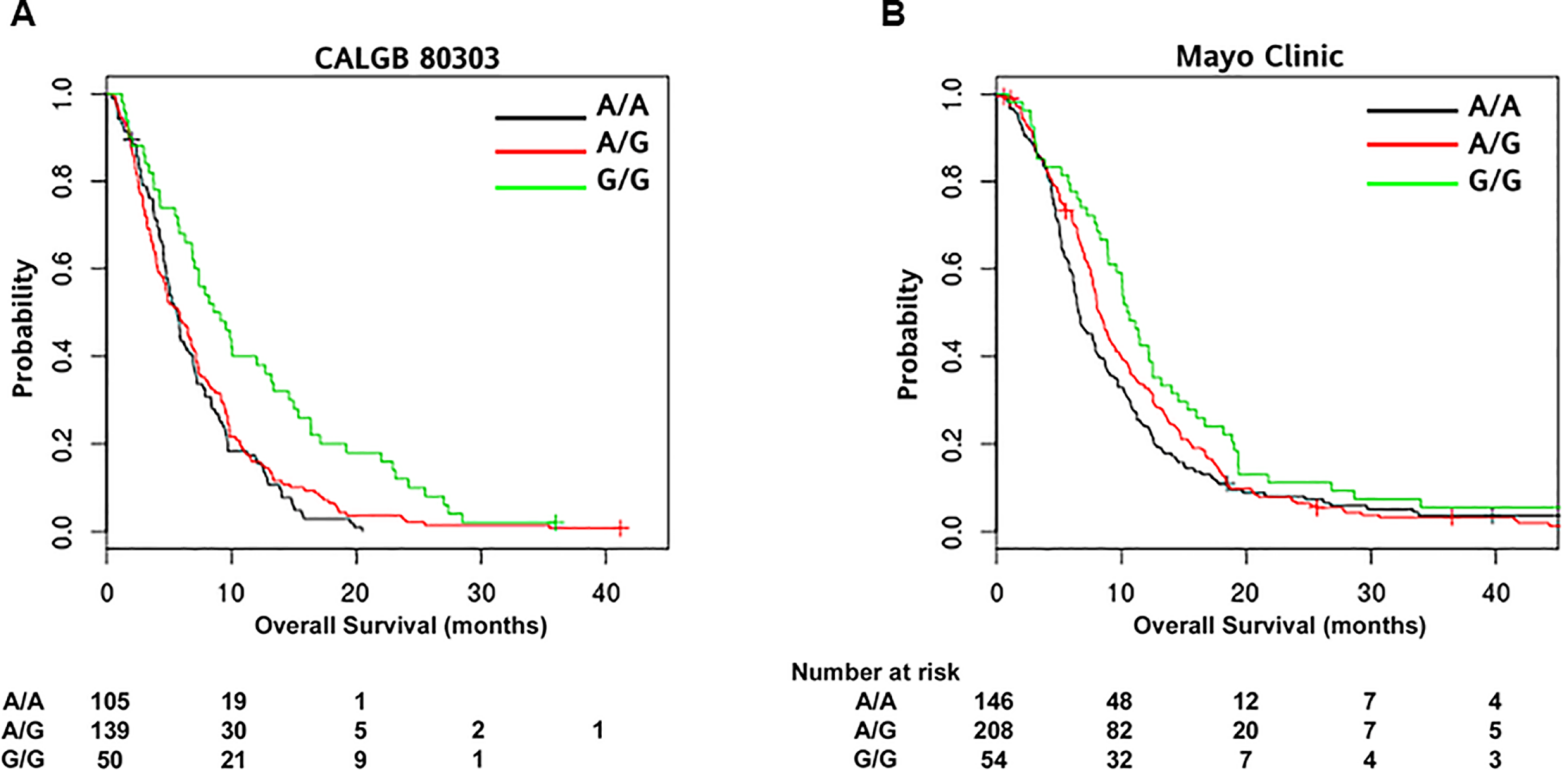
**Kaplan-Meier plots of the association between *VDR* rs2853564 and OS in patients from the CALGB 80303 (A) and Mayo Clinic (B).** rs2853564 is an A>G change, and the survival curves for each genotype are reported. Tables indicate the number of patients who were alive and at risk of death at each time point after the diagnosis of pancreatic cancer.

*VDR* rs2853564 was associated with OS in both the CALGB 80303 patients (HR 0.74, 95% CI 0.63–0.87, p = 0.0002) and the Mayo Clinic patients (hazard ratio, HR 0.81, 95% confidence intervals, CI 0.70–0.94, p = 0.0059) (Table 2). In the CALGB 80303 patients, median OS was 8.9 (95% CI 6.9–13.4), 5.9 (95% CI 4.5–7.2), and 5.7 (95% CI 4.8–6.9) months, respectively (Fig 1). When the data from both studies were combined, the association between rs2853564 and OS resulted in an HR of 0.78 (95% CI 0.70–0.87, p = 6.1*e^-6^, unadjusted data). Of the Mayo Clinic patients, those with the GG variant genotype had a median OS of 10.6 months (95% CI 9.0–13.2) compared to 8.2 months (95% CI 7.8–9.3) in AG patients and 6.6 months (95% CI 6.1–8.0) in AA patients.

### Interaction between VDR rs2853564 and 25(OH)D levels on OS

**Fig 2:**
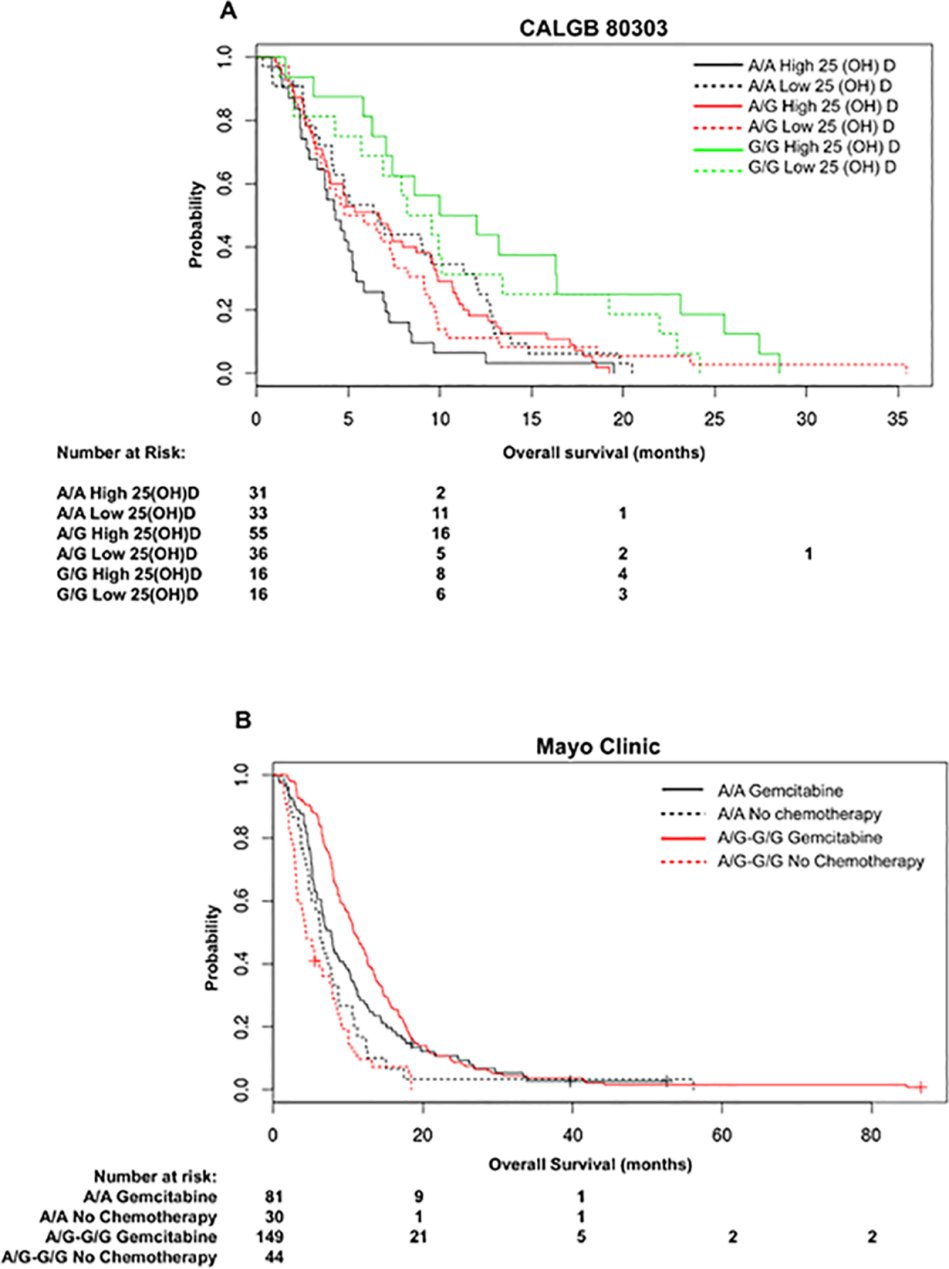
**Kaplan-Meier plots of the association between *VDR* rs2853564 and OS stratified by the serum baseline levels of 25-hydroxyvitamin D (25(OH)D) (A) and gemcitabine treatment (B). (A)** The median (95% CI) OS (months) values stratified by the level of 25(OH)D (high vs. low) and rs2853564 genotypes (AA, AG, GG) are as follows: AA/high (n = 31): 4.3 (95% CI 3.3–5.9), AA/low (n = 33): 6.6 (95% CI 4.2–12.0), AG/high (n = 55): 6.7 (95% CI 4.0–9.6), AG/low (n = 36): 5.3 (95% CI 3.6–8.3), GG/high (n = 16): 11.0 (95% CI 7.1–25.5), GG/low (n = 16): 8.9 (95% CI 5.7–22.0). **(B)** The median (95% CI) OS (months) values stratified by gemcitabine vs. no chemotherapy and rs2853564 genotypes (AA vs. AG+GG), are as follows: AA/gemcitabine (n = 81): 7.7 (95% CI 6.4–10.2), AA/no chemotherapy (n = 30): 6.3 (95% CI 4.8–8.7), AG+GG/gemcitabine (n = 149): 10.8 (95% CI 9.6–12.5), AG+GG/no chemotherapy (n = 44): 4.4 (95% CI 3.2–7.9). Tables indicate the number of patients who were alive and at risk of death at each time point after the diagnosis of pancreatic cancer.

We aimed to identify whether the effect of rs2853564 on OS was modulated by levels of circulating vitamin D. Hence, we compared patient genotype and serum levels of 25(OH)D with OS. The baseline serum levels of 25(OH)D, the major circulating metabolite of vitamin D and the best indicator of vitamin D supply, were measured in a subset (n = 187) of the CALGB 80303 patients [8]. Serum levels of 25(OH)D were unavailable from the Mayo Clinic cohort. Patients with both the rs2853564 GG genotype and high 25(OH)D levels had the longest OS (median 11.0 months, 95% CI 7.1–25.5) relative to the other patients (p = 0.0079 for interaction) (Fig 2A). A clear genotype-dose effect was observed for the group with high 25(OH)D, but the same gradient effect was not observed for the group with low 25(OH)D.

### Interaction between VDR rs2853564 and gemcitabine treatment on OS

All patients in CALGB 80303 were treated with gemcitabine. However, in the Mayo Clinic cohort, 230 patients received gemcitabine and 74 patients received no chemotherapy. Hence, for the Mayo Clinic patients, we tested the hypothesis that the association between rs2853564 and OS might change in relation to gemcitabine treatment. Due to the small number of patients in the group receiving no chemotherapy, the analysis was conducted combining the AG genotype with the GG genotype. The longest OS was observed for the patients with the AG+GG genotypes and gemcitabine treatment (median 10.8 months, 95% CI 9.6–12.5). Patients with the AA genotype treated with gemcitabine had shorter OS (7.7 months, 95% CI 6.4–10.2) (p = 0.024 for interaction) (Fig 2B).

### Function of VDR rs2853564 in luciferase reporter assays

**Fig 3:**
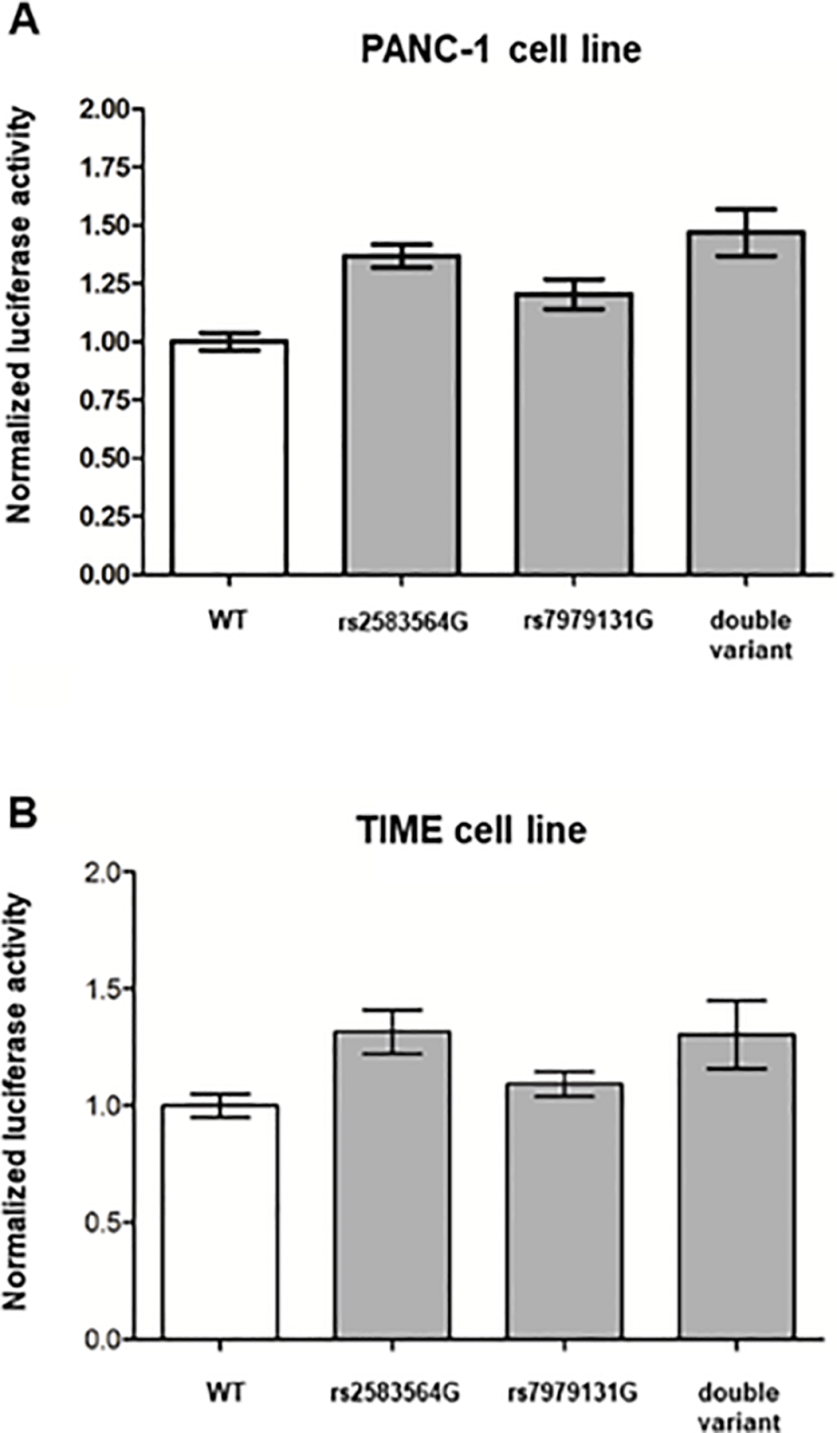
Luciferase assays of *VDR* rs2853564 and rs7979131 in two human cell lines. Luciferase assays of reporter gene constructs for rs2853564 (A>G) and rs7979131 (T>G) were performed in human pancreatic carcinoma cells (PANC-1) and telomerase-immortalized human microvascular endothelial (TIME) cells. Luciferase activity was determined as a ratio of *Firefly* to *Renilla* luciferase activity, normalized to the reference sequence construct (reported as wild-type, WT, in the graph).

Luciferase assays were conducted in PANC-1 and TIME cell lines to test whether *VDR* rs2853564 and rs7979131 affect transcriptional activity. For rs2853564 (A>G), the G allele increased luciferase activity (p = 6.5*10^−4^). For rs7979131 (T>G), there was no evidence that the G allele increased luciferase activity (p = 0.36). Additionally, the double variant construct did not show increased luciferase activity beyond that of the rs2853564 variant (p = 0.24 for interaction) (Fig 3).

### EMSA of VDR variants

Bioinformatics analysis of predicted transcription binding sites in the proximity of rs2853564 and rs7979131 has shown putative binding of IRF4, SPI1, and CTCF (S2 Table). To confirm these predictions, we performed EMSA to provide evidence of transcription factor binding. We also tested the differential effect of the reference and variant alleles on binding. IRF4 preferentially bound to the rs2853564 A allele rather than the G allele (S2A Fig). There was no evidence of differential binding of SPI1 between the A and G alleles of rs2853564 (S2B Fig). CTCF did not bind to either allele of rs7979131 (results not shown).

### VDR rs2853564 and association with VDR mRNA expression

**Fig 4:**
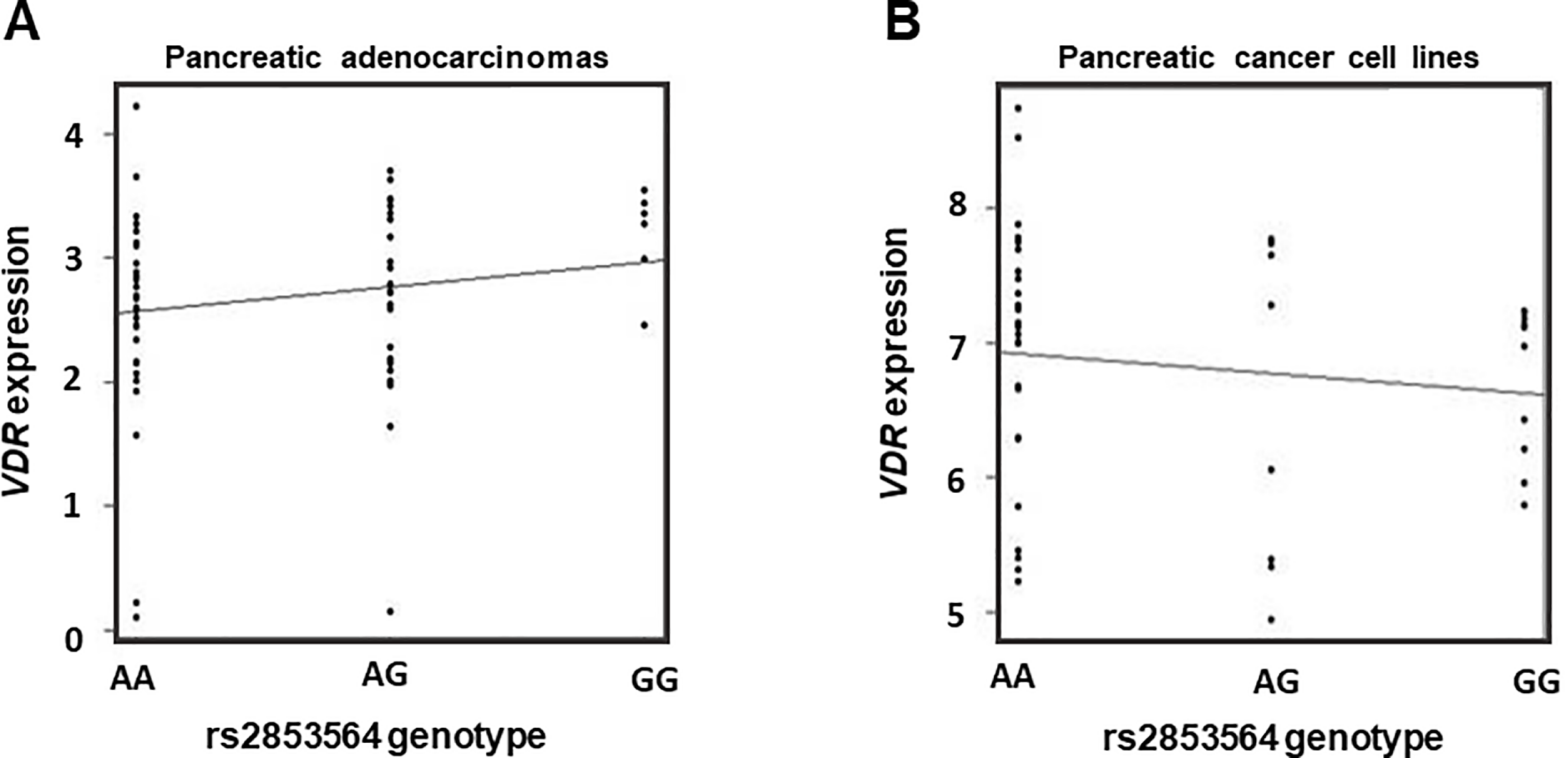
**Analysis of the association between *VDR* rs2853564 and *VDR* mRNA expression in A) pancreatic adenocarcinomas (n = 66) and B) CCLE pancreatic cancer cell lines (n = 44). A)** Genotypes for rs2853564 were determined by a TaqMan SNP genotyping assay. Gene expression data for *VDR* was derived from Agilent gene expression microarrays normalized for intra-array dye bias. Association between genotype and *VDR* mRNA expression was assessed using an additive genetic model in a linear regression. The removal of three low samples (2 AA and 1 AG) did not affect the result (p>0.05). **B)** rs2853559 instead of rs2853564 has been used in this analysis as rs2853559 is in almost complete LD (r^2^ = 0.9652) with rs2853564. In the figure, we report the genotypes of rs2853564 and their association with *VDR* expression. Genotype and *VDR* expression data were obtained from the CCLE, as described in the Supplementary Methods. *VDR* expression data were normalized using a robust multi-array average. Association between genotype and *VDR* mRNA expression was assessed using an additive genetic model in a linear regression.

Based upon the observed effect of rs2853564 in the luciferase assay, we tested whether rs2853564 might affect the mRNA expression of *VDR* in human pancreatic cancer tissues and cell lines. There was no statistically significant association between rs2853564 and *VDR* mRNA levels in either 66 pancreatic cancer tissues from an independent cohort (p = 0.194) or 44 human pancreatic cancer cell lines (p = 0.388) (Fig 4A and 4B, respectively).

## Discussion

In the present study, the results of two genomic screens of germline DNA variation led to the investigation of *VDR* as a potential genetic determinant of the survival of pancreatic cancer patients.

In models of pancreatic cancer, the biological properties of vitamin D and its receptor are well established [10–14] How such properties affect the clinical progression of pancreatic cancer patients is poorly understood. This paper is providing, for the first time, the clinical and experimental evidence that germline genetic variation in *VDR* might impact survival. The presence of a common genetic variant in *VDR* is associated with improved survival in two clinical studies. We demonstrate that this variant is functional, increasing the transcriptional efficiency of *VDR*. The clinical association is further potentiated by positive interaction with 25(OH)D levels and gemcitabine treatment. In addition to the *VDR* variant, this paper reports the association with nine other variants (Table 2) that could be considered for further investigation. The present study focuses on the downstream analyses of a variant in *VDR*.

*VDR* codes for the receptor of the active form of vitamin D, i.e. 1,25-dihydroxyvitamin D3. Preclinical studies indicate that vitamin D analogs halt progression through the cell cycle, induce apoptosis, and stop or slow the growth of pancreatic cancer in vivo [15–18]. It also potentiates the antitumor activities of a number of cytotoxic agents including gemcitabine [16]. The role of vitamin D on the prevention of pancreatic cancer is still unclear (for a review, see [19]).

In this study, two independent genomic screens of germline DNA variation in advanced pancreatic cancer patients selected *VDR* as a novel gene associated with patient survival. Patients with the rs2853564 GG genotype of *VDR* have increased median OS as compared to the AA (“wild-type”) genotype. The *VDR* variant is an intronic A>G change, with an allele frequency of about 40%, indicating its relevance to a substantial proportion of patients. The association between the *VDR* variant and OS in the Mayo Clinic cohort did not pass the threshold for statistical significance after Bonferroni correction, and this could be a limitation of these results, in addition to the limited sample size of the Mayo Clinic cohort. These aspects do not eliminate the possibility of the clinical relevance of our findings. As an example, a *WWOX* variant that did not pass statistical significance after correction for multiple testing in CALGB 80303 [7] has been recently associated with OS in another study in pancreatic cancer patients, and the association has been supported by experimental evidence [20].

Prior to this study, rs2853564 was a variant of unknown significance. In the luciferase assays, the G allele of rs2853564 determines an increase in transcriptional activity relative to the A allele in human pancreatic cancer and endothelial cell lines. Increased cellular transcription of *VDR* by the G allele of rs2853564 and its association with improved OS correspond with the well-established protective functions of *VDR* on cancer cell proliferation and differentiation described above. EMSA also indicated the molecular function of rs2853564 is mediated by an altered binding of IRF4 to *VDR*.

Primary tumor tissue was unavailable from the patients in these studies, thus we were limited in our ability to determine whether this variant might increase *VDR* expression in pancreatic cancer cells, stromal cells, or other cellular components of the tumor microenvironment. An association between rs2853564 and *VDR* mRNA levels was not detected in either resected tumors from a separate patient cohort or pancreatic cancer cell lines (Fig 4). Recent evidence suggests that genetic variation in *VDR* might affect VDR function in the microenvironment. It has been shown that the activation of *VDR* by one of its ligands in a pancreatic tumor murine model resulted in reduced tumor fibrotic content, improved vasculature, and increased delivery of gemcitabine, with improved therapeutic efficacy [13]. Combined with our clinical data, we postulate that changes in *VDR* expression and function in the tumor stroma determined by rs2853564 might affect the clinical course of pancreatic cancer. More mechanistic confirmatory studies are needed to further corroborate this hypothesis.

Due to the observed beneficial effects of endogenous levels of vitamin D for survival in pancreatic cancer, [11] we hypothesized that both the *VDR* genotype status and levels of baseline vitamin D contribute to patient survival. In a stratified analysis (Fig 2A), the association of high 25(OH)D levels with OS is highly dependent upon the rs2853564 genotype. In the group of patients with low 25(OH)D levels, such dependency is less evident, suggesting that rs2853564 might be clinically relevant primarily in the presence of adequate vitamin D supply.

In vivo animal data show an increased tumor penetration of gemcitabine and increased therapeutic efficacy when VDR is activated [13]. We, therefore, tested the effect that *VDR* rs2853564 on OS might be further potentiated by gemcitabine treatment. In the Mayo Clinic cohort, the G allele of rs2853564 interacts with gemcitabine treatment, and patients with the AG+GG genotype have improved survival when treated with gemcitabine vs. patients with the AA genotype (Fig 2B). Although these analyses are exploratory, they provide the opportunity to shed light on whether the *VDR* variant might associate with changes in OS, according to either baseline vitamin D serum levels or treatment with gemcitabine.

Together, the clinical, genetic, and experimental results from this study point toward genetic variation in *VDR* as a novel determinant of the outcome of pancreatic cancer patients treated with chemotherapy. The interplay among *VDR* rs2853564, drug treatment, and vitamin D supply should be investigated in pancreatic cancer patients in the context of clinical trials of vitamin D or its analogs combined with chemotherapy.

## Supporting information

S1 Table Bioinformatics analysis of *VDR* SNPs. The linkage disequilibrium (LD) r^2^ is in relation to rs2853564 in Europeans from the 1000 Genome Project. The SNP region is relative to the *VDR* gene. The SNP in bold (rs2853564) is the variant associated with OS in both studies (Fig 1). rs7979131 was also tested in luciferase assays, similar to rs2853564 (Fig 3). RegulomeDB score represents the evidence that each SNP functions in a regulatory role (1-strong evidence, 6-weak evidence). ENCODE data includes experimental information for ChiP-seq and DNase I sensitivity, as well as transcription factor binding motifs that were identified using a combination of computational and experimental data.
(DOCX)

S2 Table Predicted transcription factor binding sites (underlined) relative to the position of rs2853564 and rs7979131 in intron 2 of *VDR*. (DOCX)

S3 Table Patient demographics of 66 pancreatic adenocarcinomas. (DOCX)

S1 Fig CONSORT chart. (DOCX)

S2 Fig Electrophoretic mobility shift assays (EMSA) of *VDR* SNPs. EMSA using biotinylated DNA probes of rs2853564 A (reference) and G (variant) alleles. Commercially available cell lysates overexpressing (A) IRF4 or (B) SPI1 transcription factors were incubated with biotinylated DNA probes containing the rs2853564 G (lanes 5–7) or A (lanes 8–10) alleles.
(DOCX)
