# Supplementary material for: The vitamin D receptor gene as a determinant of survival in pancreatic cancer patients: Genomic analysis and experimental validation
Source: PLoS One. 2018 Aug 14;13(8):e0202272. doi: 10.1371/journal.pone.0202272 (PMC6091939; doi:10.1371/journal.pone.0202272)
Supplement: S1 Table — The linkage disequilibrium (LD) r2 is in relation to rs2853564 in Europeans from the 1000 Genome Project. The SNP region is relative to the VDR gene. The SNP in bold (rs2853564) is the variant associated with OS in both studies (Fig 1). rs7979131 was also tested in luciferase assays, similar to rs2853564 (Fig 3). RegulomeDB score represents the evidence that each SNP functions in a regulatory role (1-strong evidence, 6-weak evidence). ENCODE data includes experimental information for ChiP-seq and DNase I sensitivity, as well as transcription factor binding motifs that were identified using a combination of computational and experimental data. (DOCX) [file pone.0202272.s001.docx]

**S1 Table. Bioinformatics analysis of *VDR* SNPs.** The linkage disequilibrium (LD) r^2^ is in relation to rs2853564 in Europeans from the 1000 Genome Project. The SNP region is relative to the *VDR* gene. The SNP in bold (rs2853564) is the variant associated with OS in both studies (Figure 1). rs7979131 was also tested in luciferase assays, similar to rs2853564 (Figure 3). RegulomeDB score represents the evidence that each SNP functions in a regulatory role (1-strong evidence, 6-weak evidence). ENCODE data includes experimental information for ChiP-seq and DNase I sensitivity, as well as transcription factor binding motifs that were identified using a combination of computational and experimental data.

| **SNP** | **LD (r^2^)** | **Region** | **RegulomeDB score** |  | **ENCODE data** | |  | |  |
| --- | --- | --- | --- | --- | --- | --- | --- | --- | --- |
|  |  |  |  | **ChIP-seq** | | **Transcription factor binding motifs** | | **DNase I sensitivity region** |  |
| **rs2853564** | - | Intronic | 5 | IRF4, SPI1, BATF | | - | | - | |
| rs1989969 | 0.99 | Intronic | no data | - | | - | | - | |
| rs7965266 | 0.98 | Intronic | 5 | - | | MECOM | | Yes | |
| rs7965274 | 0.98 | Intronic | 5 | - | | - | | Yes | |
| rs7979131 | 0.97 | Intronic | 2b | CTCF, RAD21 | | GATA-2, GATA-3 | | Yes | |
| rs2853559 | 0.93 | Intronic | no data | - | | - | | - | |
| rs7965943 | 0.788 | Intronic | 6 | - | | NANOG, SOX4, | | - | |
| rs2254210 | 0.6 | Intronic | 5 | - | | - | | Yes | |
| rs3922882 | 0.58 | Intronic | 6 | - | | COUPTF | | - | |
| rs4760648 | 0.5 | Intronic | 5 | - | | - | | Yes | |
| rs4760658 | 0.41 | Intronic | 5 | - | | - | | Yes | |
| rs11168292 | 0.4 | Intronic | no data | - | | - | | - | |
| rs11168293 | 0.4 | Exonic | 6 | - | | NEUROD | | - | |
| rs3890733 | 0.4 | Intronic | no data | - | | - | | - | |
| rs3890734 | 0.4 | Intronic | no data | - | | - | | - | |
| rs2853561 | 0.38 | Intronic | no data | - | | - | | - | |
| rs11168284 | 0.37 | Intronic | 4 | SPI1, POLR2A | | - | | Yes | |
| rs11168283 | 0.36 | Intronic | 4 | FOXA1 | | - | | Yes | |
| rs11168287 | 0.31 | Intronic | 4 | MAFF, MAFK | | - | | Yes | |
| rs10875696 | 0.26 | 5' flanking | no data | - | | - | | - | |
| rs11614332 | 0.26 | 5' flanking | 4 | - | | CDX2, HNF4A | | Yes | |
| rs7310552 | 0.26 | 5' flanking | 5 | - | | - | | Yes | |
| rs11168297 | 0.25 | 5' flanking | 6 | - | | ZFX, PITX2 | | - | |
| rs4516035 | 0.25 | 5' flanking | no data | - | | - | | - | |
| rs7139166 | 0.25 | 5' flanking | 5 | - | | RBP-JKAPPA, STAF, ZNF143 | | Yes | |
| rs7296550 | 0.23 | 5' flanking | 6 | - | | - | | - | |
