# Supplementary material for: The vitamin D receptor gene as a determinant of survival in pancreatic cancer patients: Genomic analysis and experimental validation
Source: PLoS One. 2018 Aug 14;13(8):e0202272. doi: 10.1371/journal.pone.0202272 (PMC6091939; doi:10.1371/journal.pone.0202272)
Supplement: S2 Table — (DOCX) [file pone.0202272.s002.docx]

**S2 Table. Predicted transcription factor binding sites (underlined) relative to the position of rs2853564 and rs7979131 in intron 2 of *VDR*.**

| **SNP** | **Transcription factor** | **Transcription factor motifs** | **Genomic region** |
| --- | --- | --- | --- |
| **rs2853564** | IRF4 | ATTTCTGCAACCCTAAGCC**A/G**TGGACA**CCTTTC**ACTTCCAC | chr12: 48278494-48278499 |
| **rs2853564** | SPI1 | ATTTCTGCAACCCTAAGCC**A/G**TGGACACCTTTCA**CTTCC**AC | chr12: 48278501-48278505 |
| **rs7979131** | CTCF | ATGTCCACAGTAAAAC**CCAAT/GAG**ATAACATTCAGCTCTGAA | chr12: 48280451-48280457 |
