# Supplementary material for: The vitamin D receptor gene as a determinant of survival in pancreatic cancer patients: Genomic analysis and experimental validation
Source: PLoS One. 2018 Aug 14;13(8):e0202272. doi: 10.1371/journal.pone.0202272 (PMC6091939; doi:10.1371/journal.pone.0202272)
Supplement: S3 Table — (DOCX) [file pone.0202272.s003.docx]

**S3 Table. Patient demographics of 66 pancreatic adenocarcinomas.**

| **Sample Size** |  | 66 |
| --- | --- | --- |
| **Sex** | Male | 26 (39.4%) |
|  | Female | 40 (60.6%) |
| **Age** | Mean (95%CI) | 68.9 (66.0, 71.8) |
| **Ethnicity** | Caucasian | 50 (75.7%) |
|  | African American | 5 (7.6%) |
|  | Unknown | 11 (16.7%) |
| **Stage** | IA | 1 (1.5%) |
|  | IB | 5 (7.6%) |
|  | IIA | 18 (27.3%) |
|  | IIB | 37 (56.0%) |
|  | Unknown | 5 (7.6%) |
