# Supplementary figures and images for: The vitamin D receptor gene as a determinant of survival in pancreatic cancer patients: Genomic analysis and experimental validation
Source: PLoS One. 2018 Aug 14;13(8):e0202272. doi: 10.1371/journal.pone.0202272 (PMC6091939; doi:10.1371/journal.pone.0202272)

**S1 Fig. CONSORT chart.**


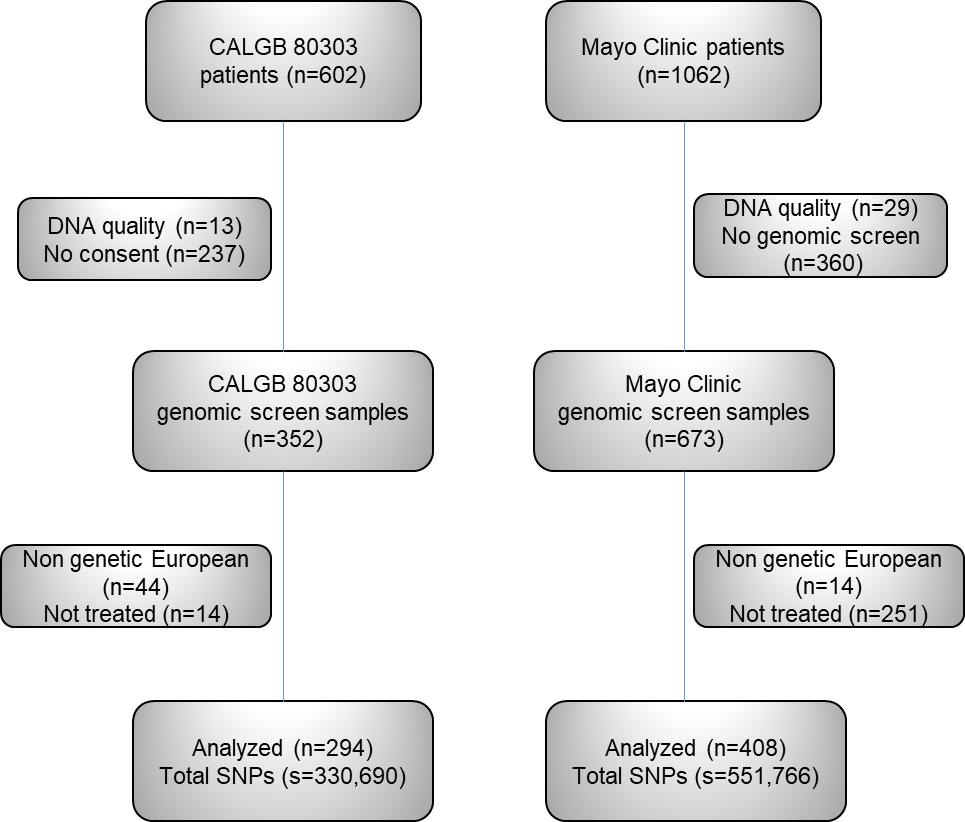

Supplement: S1 Fig — (DOCX) [file pone.0202272.s004.docx]
