# Supplementary material for: The vitamin D receptor gene as a determinant of survival in pancreatic cancer patients: Genomic analysis and experimental validation
Source: PLoS One. 2018 Aug 14;13(8):e0202272. doi: 10.1371/journal.pone.0202272 (PMC6091939; doi:10.1371/journal.pone.0202272)
Supplement: S2 Fig — EMSA using biotinylated DNA probes of rs2853564 A (reference) and G (variant) alleles. Commercially available cell lysates overexpressing (A) IRF4 or (B) SPI1 transcription factors were incubated with biotinylated DNA probes containing the rs2853564 G (lanes 5–7) or A (lanes 8–10) alleles. (DOCX) [file pone.0202272.s005.docx]

**S2 Fig. Electrophoretic mobility shift assays (EMSA) of *VDR* SNPs.** EMSA using biotinylated DNA probes of rs2853564 A (reference) and G (variant) alleles. Commercially available cell lysates overexpressing (A) IRF4 or (B) SPI1 transcription factors were incubated with biotinylated DNA probes containing the rs2853564 G (lanes 5-7) or A (lanes 8-10) alleles.

**
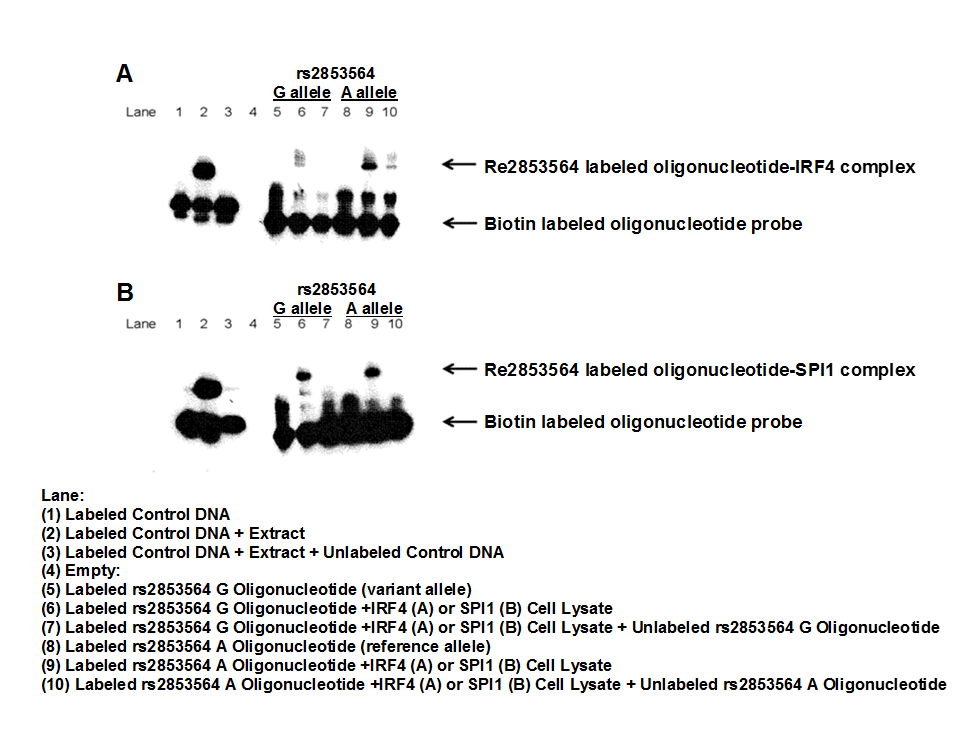
**
